# Interferon-γ and Tumor Necrosis Factor-α Polarize Bone Marrow Stromal Cells Uniformly to a Th1 Phenotype

**DOI:** 10.1038/srep26345

**Published:** 2016-05-23

**Authors:** Ping Jin, Yuanlong Zhao, Hui Liu, Jinguo Chen, Jiaqiang Ren, Jianjian Jin, Davide Bedognetti, Shutong Liu, Ena Wang, Francesco Marincola, David Stroncek

**Affiliations:** 1 Cell Processing Section, Department of Transfusion Medicine, Clinical Center, National Institutes of Health, Bethesda, Maryland 20892 USA; 2 Center for Human Immunology (CHI), National Institutes of Health, Bethesda, MD, 20892, USA; 3 Research Branch, Sidra Medical and Research Center, Doha, Qatar

## Abstract

Activated T cells polarize mesenchymal stromal cells (MSCs) to a proinflammatory Th1 phenotype which likely has an important role in amplifying the immune response in the tumor microenvironment. We investigated the role of interferon gamma (IFN-γ) and tumor necrosis factor alpha (TNF-α), two factors produced by activated T cells, in MSC polarization. Gene expression and culture supernatant analysis showed that TNF-α and IFN-γ stimulated MSCs expressed distinct sets of proinflammatory factors. The combination of IFN-γ and TNF-α was synergistic and induced a transcriptome most similar to that found in MSCs stimulated with activated T cells and similar to that found in the inflamed tumor microenvironment; a Th1 phenotype with the expression of the immunosuppressive factors IL-4, IL-10, CD274/PD-L1 and indoleamine 2,3 dioxygenase (IDO). Single cell qRT-PCR analysis showed that the combination of IFN-γ and TNF-α polarized uniformly to this phenotype. The combination of IFN-γ and TNF-α results in the synergist uniform polarization of MSCs toward a primarily Th1 phenotype. The stimulation of MSCs by IFN-γ and TNF-α released from activated tumor infiltrating T cells is likely responsible for the production of many factors that characterize the tumor microenvironment.

Mesenchymal stromal cells (MSCs) are multipotent cells that play a central role in tissue regeneration, wound healing, and maintenance of tissue homeostasis^1^,^2^,^3^. MSCs are found in almost every tissue including the bone marrow, where they are known as bone marrow stromal cells (BMSCs). The mechanism by which MSCs exert their immunomodulatory functions is complex, but has been shown to be dependent upon the cross-talk between MSCs and immune cells and involves both direct contact and the release of soluble factors^4^.

We have been investigating the role of MSCs in the tumor microenvironment. Previously, we used BMSCs and tumor infiltrating lymphocytes (TIL) co-cultured with melanoma cells as a model to study changes in the tumor microenvironment. By using such an approach we have shown that when MSCs are co-cultured with melanoma-activated TIL, the MSCs are polarized to a Th1-like phenotype which was associated with marked pro-inflammatory changes^5^. We found that the Th1 phenotype was induced by soluble factors produced by activated TIL. While activated TIL predominantly induced the transcription of proinflammatory MSC genes, they also triggered the expression of immune suppressive molecules such as indoleamine 2,3 dioxygenase (IDO). Furthermore, our results suggested that the cytokines IFN-γ and TNF-α play a critical role in MSC polarization, but other factors secreted by the TIL or tumor cells used in our model might have contributed to the observed MSC phenotypical changes.

As co-culture with activated TIL can be technical challenging, we assessed whether the detected changes following TIL co-culture could be reproduced by treating MSCs with IFN-γ, TNF-α or the combination of both cytokines. In our previous study we noticed that activated TILs co-expressed both IL-12 (a Th1-related cytokine) and IDO. We therefore employed single cell transcriptomic analysis to assess the scope of divergence or convergences of MSC polarization following cytokine stimulation.

## Results

### IFN-γ and TNF-α Induce the Expression of Proinflammatory Genes in MSCs

**Figure 1:**
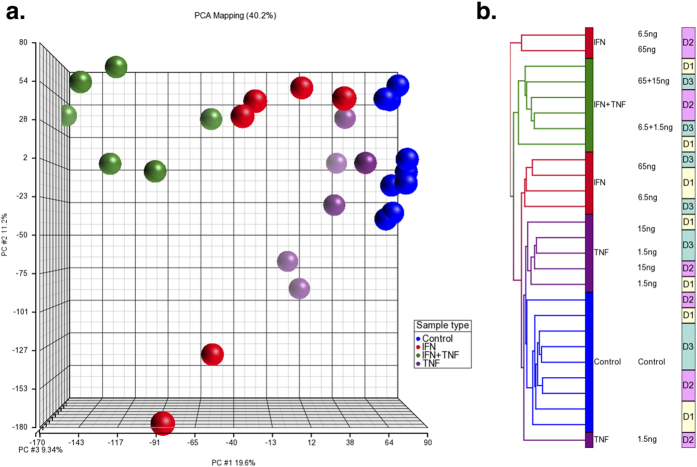
Global gene expression analysis of the MSCs stimulated with IFN-γ alone, TNF-α alone or the combination of IFN-γ plus TNF-α (IFN-γ + TNF-α). MSCs from 3 healthy subjects were stimulated with IFN-γ (6.5 ng or 65 ng), TNF-α (1.5 ng or 6.5 ng) or IFN-γ + TNF-α (6.5 ng and 1.5 ng or 65 ng and 15 ng). The entire data set was analyzed by PCA (**a**) and unsupervised hierarchical clustering analysis (**b**). MSCs stimulated with IFN-γ are shown in red, TNF-α in purple and IFN-γ + TNF-α in green. Control MSCs are shown in blue.

After 48 hours of stimulation of MSCs with IFN-γ, TNF-α or the combination of IFN-γ plus TNF-α (IFN-γ + TNF-α), the MSCs were analyzed by gene expression profiling. The results showed that unique MSC gene expression patterns were induced by the three conditions. Principle Component Analysis (PCA) based on the whole transcriptome separated the MSCs stimulated with IFN-γ + TNF-α from the control MSCs, while MSCs stimulated with IFN-γ alone and TNF-α alone were grouped between control MSCs and those cultured with IFN-γ + TNF-α (See Fig. 1a). The main factor influencing MSC segregation was the treatment (i.e. IFN-γ, TNF-α or their combination), while dose had a minor effect.

Unsupervised hierarchical clustering analysis revealed that the MSCs formed distinct clusters based on stimulation type except for three samples from one donor, implying a certain influence of donor-associated factors (See Fig. 1b). Two samples from this donor were stimulated with IFN-γ and another was stimulated with TNF-α. However, combined treatment eliminated such donor-dependent variability. The MSC samples were separated into two major groups; one group included all of the IFN-γ + TNF-α MSCs and the other most of the IFN-γ alone, TNF-α alone and control MSC samples (See Fig. 1b). Within the cluster with the mixed MSC types, the samples formed 3 groups; one with most of the IFN-γ alone MSCs, one with most of the TNF-α alone MSCs and one with all of the control MSCs. These results showed that MSCs changed their gene expression patterns upon stimulation with inflammatory cytokines IFN-γ and TNF-α and that IFN-γ and TNF-α had synergistic effects.

### IFN-γ and TNF-α Stimulation Polarizes MSCs to a Proinflammatory Phenotype

**Figure 2:**
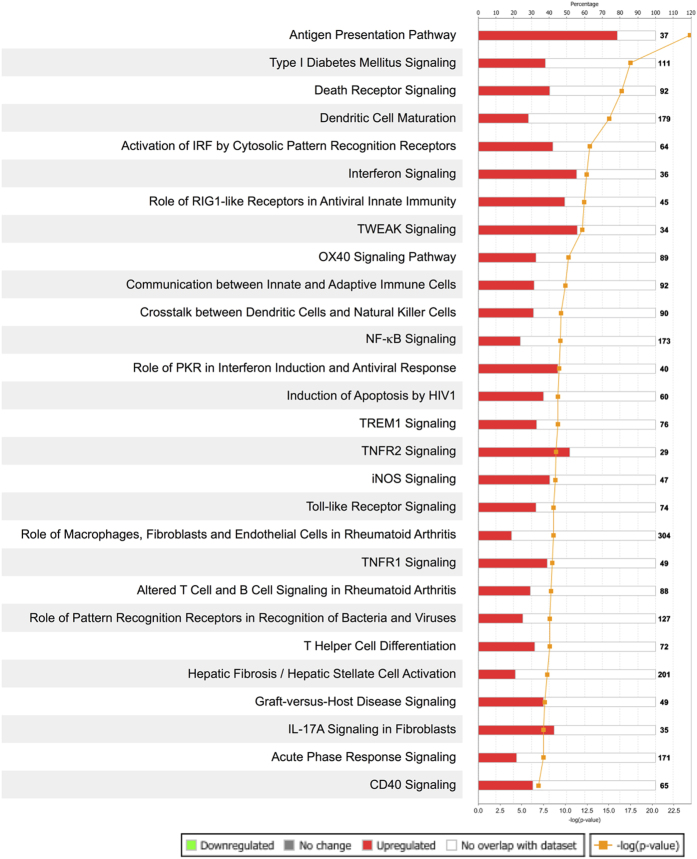
Ingenuity Pathway Analysis (IPA) of genes expressed by MSCs stimulated with IFN-γ and TNF-α. A total of 6994 genes were differentially expressed between MSCs from 3 subjects stimulated with IFN-γ + TNF-α (6.5 ng and 1.5 ng or 65 ng and 15 ng) and control MSCs (p and FDR ≤ 0.05). The 6994 differentially expressed genes were analyzed by IPA. The pathways with the most over represented genes are shown.

Since IFN-γ and TNF-α have a synergistic effect on MSCs stimulation, we focused our analysis on the IFN-γ + TNF-α MSC group of samples. We assessed transcription activation in IFN-γ + TNF-α stimulated MSCs using a list of 6994 genes that were differentially expressed among IFN-γ + TNF-α MSC and control samples (p < 0.05 and FDR < 0.05). IPA revealed that the most enriched pathways in the IFN-γ + TNF-α MSC samples included many immune related pathways such as Antigen Presentation Pathway, Dendritic Cell Maturation, Interferon Signaling and Communication between Innate and Adaptive Immune Cells (See Fig. 2). These results are consistent with those of previous analysis of the effects of activated TIL on MSCs^5^. Furthermore, there was more overlap in enriched pathways among MSCs stimulated with IFN-γ + TNF-α and MSCs co-cultured with activated TIL than with MSCs stimulated with IFN-γ alone and MSCs stimulated with TNF-α alone (Table 1).

**Figure 3:**
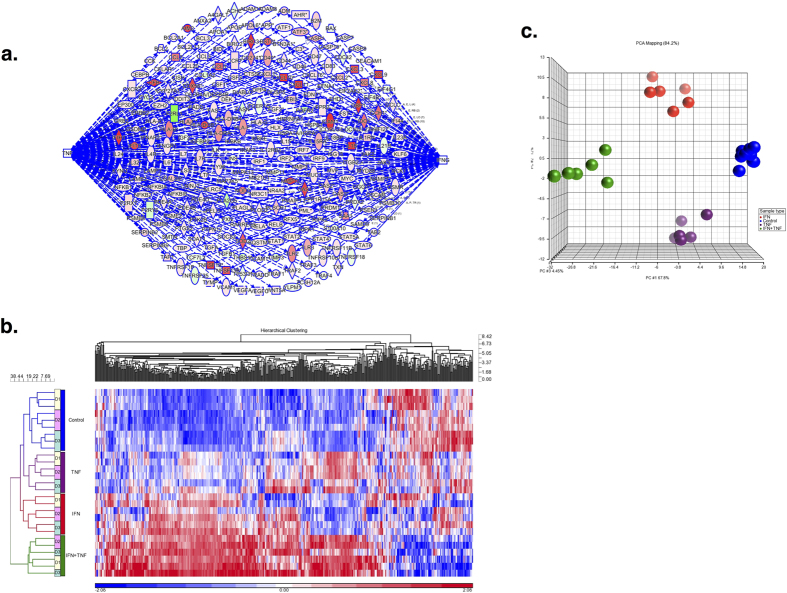
IFN and TNF genes expressed in MSCs stimulated with IFN-γ + TNF-α. (**a**) Among the 6994 genes differently expressed between IFN-γ + TNF-α stimulated and control MSCs, 311 belonged to IFN or TNF pathways identified using IPA. (**b**) Hierarchical clustering analysis of IFN- and TNF-related genes expressed by MSCs stimulated with IFN-γ + TNF-α. The expression of the 311 IFN- and TNF-related genes by MSCs from the 3 healthy subjects stimulated with IFN-γ, TNF-α or IFN-γ + TNF-α was analyzed by hierarchical clustering analysis. (**c**) PCA analysis of IFN- and TNF-related genes expressed by MSCs stimulated with IFN-γ + TNF-α. The expression of the 311 IFN- and TNF-related genes by MSCs from the 3 healthy subjects stimulated with IFN-γ, TNF-α or IFN-γ + TNF-α was analyzed by PCA. MSCs stimulated with IFN-γ are shown in red, TNF-α in purple and IFN-γ + TNF-α in green. Control MSCs are shown in blue.

We used IPA analysis to identify IFN- and TNF-related genes among the 6994 differentially expressed genes. This analysis yielded a total of 311 genes (See Fig. 3a and Supplemental Table 1). Among these 311 IFN- and TNF-related genes were many proinflammatory cytokines and chemokines including CCL5, CXCL9, CXCL10, CXCL11, IL12 and IL15. The expression of all of these genes was greater in IFN-γ + TNF-α MSC samples than the control group (Table 2). In addition, the expression of the immunosuppressive factor IDO was also increased in IFN-γ + TNF-α MSCs together with the expression of other immunosuppressive molecules (ie, CD274/PD-L1 and HLA-G). Hierarchical clustering analysis of all MSC samples using these 311 genes showed that MSCs formed distinct clusters based on the nature of cytokine stimulation (See Fig. 3b) as did PCA (See Fig. 3c). Both PCA and hierarchical clustering analysis showed that MSCs stimulated with IFN-γ or TNF-α alone formed a large cluster along with control MSCs and the differences in expression of proinflammatory factors among these groups was small compared to differences with MSCs stimulated with the combination of IFN-γ and TNF-α (Supplemental Tables 2 and 3).

The microenvironment of melanoma metastases that are likely to response to immunotherapy is characterized by the presence of lymphocytes and the expression of the inflammatory chemokines CCL2, CCL3, CCL5, CXCL9, CXCL10 and CXCL11^6^ and the immunosuppressive factors indoleamine-2,3-dioxygenase (IDO), PD-L1 and FoxP3^7^. Only the combination of IFN-γ and TNF-α was able to coordinately induce the expression of transcripts coding for the Th1 polarizing chemokines CCL5 (CCR5 ligand), CXCL9, CXCL10, and CXCL11 (CXCR3 ligands) (Table 2). The combination, however, induced the expression of the immune suppressive molecules IDO1 and CD274 (PD-L1), which were also induced by IFN-γ alone, but only weakly or not at all by TNF-α (Table 2). Interestingly, the increase in expression of these factors by MSC stimulation with IFN-γ + TNF-α was similar to the changes induced in MSCs by melanoma activated TIL^5^ (Table 2).

### Culture Supernatant Analysis Shows that the Expression of Proinflammatory Factors are Increased at the Protein Level

**Figure 4:**
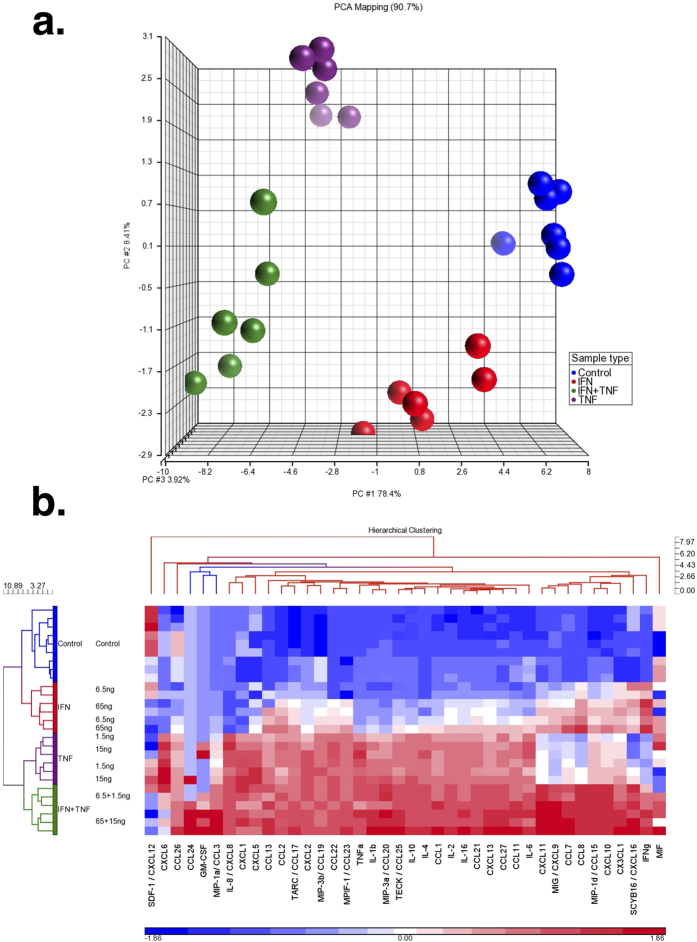
Proinflammatory cytokines and chemokines released by IFN-γ and TNF–α stimulated MSCs. MSCs from 3 healthy subjects were stimulated with IFN-γ, TNF-α or IFN-γ + TNF-α and the supernatants were analyzed by multiplex ELISA for the presence of 40 factors. (**a**) The factor levels were analyzed by PCA. Each dot represents supernatants from one sample. MSCs stimulated with IFN-γ are shown in red, TNF-α in purple and IFN-γ + TNF-α in green. Control MSCs are shown in blue. (**b**) Hierarchical clustering analysis of proinflammatory cytokines and chemokines released by IFN-γ and TNF–α stimulated MSCs.

We measured 40 soluble factors in MSC supernatants. PCA of the data showed that the expression patterns of the 40 cytokines were different among the four groups; IFN-γ, TNF-α, IFN-γ + TNF-α and control MSC supernatants (See Fig. 4a). Unsupervised hierarchical clustering analysis showed that the different stimulation conditions affected MSC secretion of cytokines and chemokines (See Fig. 4b). Most of these soluble factors were selectively induced either by IFN-γ or TNF-α. Both IFN-γ and TNF-α induced the expression of certain chemokines (e.g., CCL2, CCL13, CCL17, CCL19, CCL22, CCL23 and CXCL2). The secretion of classical Th1 CXCR3-ligand chemokines (i.e., CXCL9, CXL10, and CXCL11) was selectively induced by IFN-γ. In addition, IFN-γ induced the secretion of CCL7, CCL8, CCL15 and CXCL16. TNF-α selectively induced the production of CCL1, CCL3, CCL11, CCL20, CCL21, CCL25, CCL26, CCL27, CXCL1, CXCL5, CXCL6, CXCL8, CXCL13, IL-1B, IL-2, IL-4, IL-6, IL-10 and IL-16. In general, the induction of cytokine secretion was stronger for MSCs stimulated by the combination of IFN-γ and TNF-α than either factor alone, especially for the Th1 chemokines except for CXCL12 whose levels were only increased by TNF-α. Furthermore, the production of GM-CSF and CCL24 were uniformly induced only by MSCs stimulated with the combination of IFN-γ and TNF-α.

### Single Cell Analysis Shows that MSCs Uniformly Increase Both Proinflammatory and Immusuppressive Factor Expression

Since MSCs are heterogeneous and when stimulated with IFN-γ + TNF-α, they express both proinflammatory and immunosuppressive factors, we used single cell analysis to assess the degree of phenotypical changes across individual cells. We isolated single cells from a MSC sample from one healthy subject before and after IFN-γ + TNF-α stimulation and assessed single MSC gene expression by high throughput qRT-PCR analysis.

**Figure 5:**
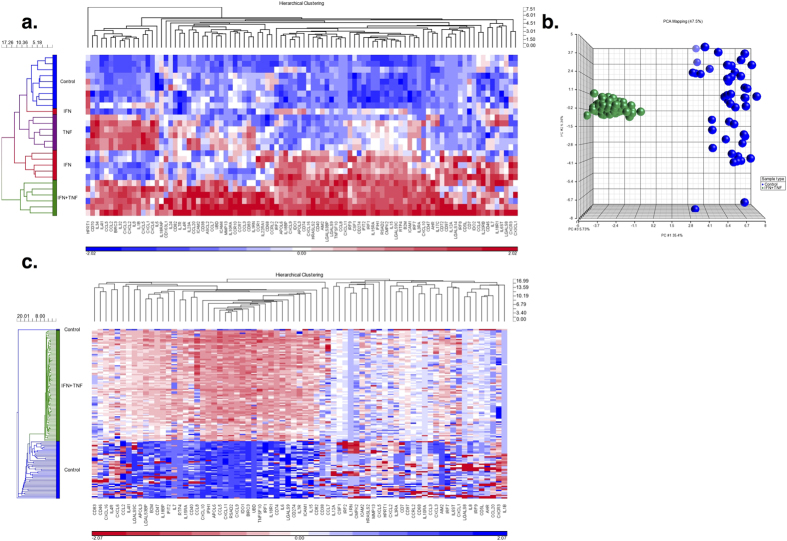
(**a**) The expression by unseparated MSCs of 93 inflammatory cytokine and chemokine genes following IFN-γ and TNF-α stimulation. MSCs from 3 healthy subjects were stimulated with IFN-γ, TNF-α or IFN-γ + TNF-α and the expression of 93 inflammatory cytokines and chemokines was evaluated by hierarchical clustering. (**b**) The expression by single MSCs of the 93 inflammatory cytokine and chemokine genes following IFN-γ + TNF-α stimulation. MSCs from one healthy subject were stimulated with IFN-γ + TNF-α and single MSCs were captured and evaluated for the expression of the 93 inflammatory cytokines and chemokines genes using single cell qRT-PCR. The results were evaluated by PCA. MSCs stimulated with IFN-γ + TNF-α are shown in green and control MSCs are shown in blue. (**c**) Hierarchical clustering analysis of the expression of 93 inflammatory cytokine and chemokine genes by single IFN-γ + TNF-α stimulated MSCs.

We analyzed in single MSCs the expression of some of the 311 IFN- and TNF-related genes. The first analysis of single MSCs used a set of 93 of the 311 IFN- and TNF-related which were inflammatory cytokine and chemokine genes and 3 housekeeping genes. Among the 93 genes were many proinflammatory factors such as CXCL9, CXCL10, CXCL11, IL12 and IL15 and immuosuppressive factors such as IDO1 and CD274 (PD-L1) (Supplemental Table 4). To evaluate the 93 gene set hierarchical clustering analysis of the IFN-γ, TNF-α and IFN-γ + INF-α stimulated unseparated MSC samples and control unseparated MSC samples from 3 subjects was performed using the expression levels of the 93 genes obtained from the global gene expression microarray data. As expected, this analysis separated the samples by stimulation type (See Fig. 5a).

Next, single MSCs that had been stimulated with IFN-γ + TNF-α were analyzed for the expression of the 93 genes using qRT-PCR. PCA of the single MSC qRT-PCR data separated all of the single MSCs stimulated with IFN-γ + TNF-α from the control single MSCs (See Fig. 5b). Strikingly, stimulated single MSCs clustered tightly together while the control unstimulated single MSCs were widely scattered. This suggests that control MSCs are a heterogeneous population, but after IFN-γ + TNF-α stimulation all MSCs were polarized to the same phenotype. Hierarchical clustering analysis of single cell qRT-PCR data revealed that some IFN-γ + TNF-α stimulated MSC transcripts had heterogeneous patterns of expression, however, the Th1 factors CXCL9, CXCL10, CXL11 and the immune suppressive molecules IDO-1 and PDL1 (CD274) were induced in virtually all cells (See Fig. 5c).

**Figure 6:**
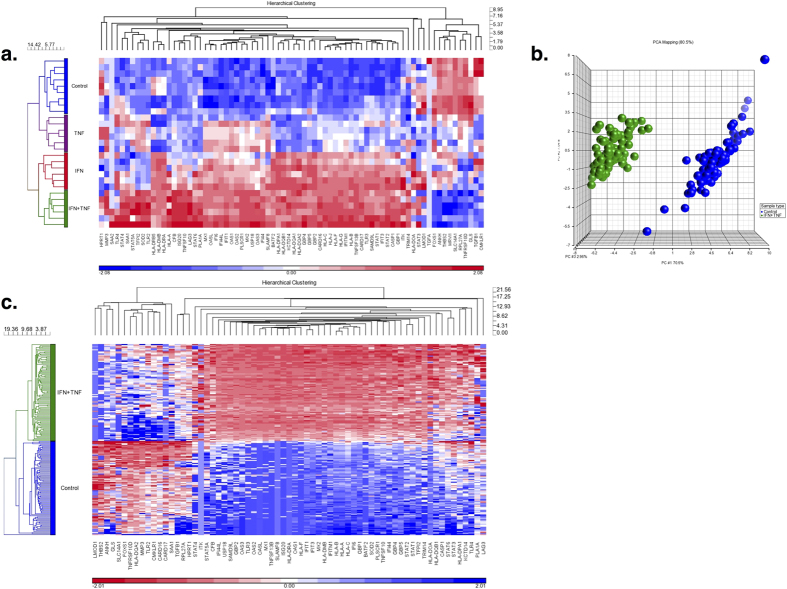
(**a**) The expression by unseparated MSCs of 73 HLA, transcription factor and toll-like receptor-related genes following IFN-γ and TNF-α stimulation. MSCs from 3 healthy subjects were stimulated with IFN-γ, TNF-α or IFN-γ + TNF-α and the expression of the 73 HLA, transcription factor and toll-like receptor-related genes was evaluated by hierarchical clustering. (**b**) The expression by single MSCs of 73 HLA, transcription factor and toll-like receptor related genes following IFN-γ + TNF-α stimulation. MSCs from one healthy subject were stimulated with IFN-γ + TNF-α and the results were subjected PCA. The MSCs stimulated with IFN-γ + TNF-α are shown in green and control MSCs in blue. (**c**) Hierarchical clustering analysis of the expression of 73 HLA, transcription factor and toll-like receptor related genes by single MSCs following IFN-γ + TNF-α stimulation.

Next, we analyzed single MSCs using a second set of 73 of the 311 IFN- and TNF- related genes that were related to Human Leukocyte Antigen (HLA), transcription factors, and toll-like receptors (TLRs) and 3 housekeeping genes (Supplemental Table 5). The results of evaluating these 73 genes using the unseparated MSC samples from 3 subjects and the global microarray gene expression data are shown in Fig. 6 (See Fig. 6a). As expected, the MSCs were separated by hierarchical clustering analysis based on stimulation type. Next, IFN-γ + TNF-α stimulated and control single MSCs were evaluated for the expression of the 73 genes by qRT-PCR. PCA of the single MSC qRT-PCR data showed that all of the single IFN-γ + TNF-α simulated MSCs again were separated from the control single MSCs (See Fig. 6b) and as with the first analysis with the set of 93 genes, all stimulated single MSCs clustered tightly together while control single MSCs were widely scattered. Hierarchical clustering analysis of single cell qRT-PCR data based on these 73 genes also confirmed that all of the single MSCs have the same gene expression pattern after IFN-γ + TNF-α stimulation (See Fig. 6c). These results showed that all of the single MSCs are polarized to the same Th1 phenotype after IFN-γ + TNF-α stimulation.

## Discussion

We have previously found that MSCs were polarized toward a Th1-like phenotype by soluble factors produced by activated TIL^5^. In this study, among the factors produced by activated TIL, we selected IFN-γ and TNF-α to investigate. We found the combination of IFN-γ plus TNF-α induced similar changes in MSCs as activated TIL including marked pro-inflammatory changes in MSCs. The Th1 MSC phenotype induced by activated TIL could not be replicated by either stimulation with IFNγ-alone or TNF-α alone, stimulation with both factors was needed.

The tumor microenvironment plays an important role in the evolution of cancer and in its response to immunotherapy. Consistently, data from gene expression profiling in humans have demonstrated that a Th1 polarized microenvironment is associated with a favorable response to various kind of immunotherapy^8^ including IL-2-based therapy^9^, adoptive therapy^10^, vaccination^11^ and immune-checkpoint inhibitors (both CTLA-4^12^ and PD-1^13^). Similarly, patients bearing a Th1-like phenotype enjoy prolonged progression free survival after a tumor is excised^14^,^15^. Importantly, following immunotherapy administration, the induction of a strong Th1 polarized response is critical for the induction of tumor rejection^8^,^9^,^12^,^16^,^17^,^18^.

This Th1 phenotype is characterized by the expression of a number of IFN-stimulated genes whose expression is orchestrated by the activation of IRF-1 and STAT-1 transcription factors. The Th1 phenotype signature overlaps with those observed during other tissue rejection phenomena such as acute graft versus host disease and allograft rejection^14^,^19^. Central in this immune constant of rejection is the activation of the Th1 CXCR3/CCR5 chemokine receptor pathways through the induction of CCL5 (CCR5 ligand), CXCL9, CXCL10 and CXCL11 (CXCR3 ligands) transcripts.

Previously, we found that melanoma-activated TIL induced MSCs to produce a wide variety of chemokines including the Th1 chemokines CCL5, CXCL9, CXCL10 and CXCL11 and the Th1 cytokine IL-12^5^. These stimulated MSCs also expressed other pro-inflammatory chemokines and cytokines that could amplify the inflammatory loop.

In this study we observed that both IFN-γ and TNF-α induced MSC gene expression of several inflammatory molecules; especially Th1-related transcripts. IFN-γ induced the expression of CXCL9 and CXCL11 while TNF-α induced the expression of the CCR5 chemokine CCL5. However, only the combination was able to induce strong and coordinated expression of the Th1 chemokines CCL5, CXCL9, CXCL10 and CXCL11 (Table 2 and Fig. 5a). Proteomic assays confirmed the transcriptomic data (See Fig. 4b). Interestingly, the secretion of CCL24 and GM-CSF were only increased in MSCs treated with both IFN-γ and TNF-α. GM-CSF has both activating and regulatory properties on T cells and a recent randomized study has shown that it is able to increase the efficacy of the CTLA-4 checkpoint inhibitor ipilimumab in patients with metastatic melanoma^20^,^21^.

While MSCs can be polarized to a proinflammatory or Th1 phenotype by IFN-γ and TNF-α, the polarized MSCs also expressed anti-inflammatory molecules such as IDO1, CD274/PDL1 and HLA-G. We used single cell transcriptomic analysis to determine if the Th1 and anti-inflammatory factors expressed by pooled MSCs populations were due to the presence of two separate cell populations. The results showed that at baseline there was considerable heterogeneity in the expression of IFN- and TNF-related genes. However, after IFN-γ plus TNF-α stimulation MSCs became more homogenous. A minority of the IFN-γ plus TNF-α stimulated MSCs showed substantial heterogeneity in gene expression, however, transcripts encoding for Th1 chemoattractant molecules (e.g., CCL5, CXCL9, CXCL10 and CXCL11) and the classical immunosuppressive molecules were strongly upregulated in all stimulated single MSCs. The lack of disjunction between Th1 response and regulatory response reflect what has been observed by profiling tumor samples. The classic view of the tumor microenvironment postulates the existence of two opposite microenvironments; one sustaining tumor growth and exemplified by the presence of regulatory T cells, and the other one promoting tumor suppression and characterized by the presence of cytotoxic T cells and Th1 cells. The current view, derived from gene expression studies across multiple human cancers suggests that the inflammatory phenotype typified by the expression of Th1 transcripts (i.e. CXCL9, CXCL10, CXCL11 and CCL5) and IFN-stimulated transcripts is also characterized by the counter-activation of suppressive mechanisms (e.g. IDO and PDL1)^12^,^22^,^23^. Tumors lacking these two characteristics are less sensitive to therapeutic immune manipulations.

In view of the relevance of the Th1 phenotype in mediating tumor rejection, the results of our present study have implications for clinical therapy. Intratumoral injection of adenoviral vectors expressing IFN-γ is being used to potentiate the effectiveness of immune therapy of melanoma^24^ and as a primary treatment of cutaneous B-cell lymphomas^25^. While these preliminary studies have been promising, our investigations suggest that the use of the combination of vectors expressing IFN-γ and TNF-α could potentiate the anti-tumor response. Some investigators have advocated the use of tumor-targeting monoclonal antibodies coupled to type I interferon molecules as cancer immunotherapy^26^. Our results suggest that antibodies coupled to both IFN-γ and TNF-α would also be effective, perhaps more so. Our results also suggest that these immunotherapies would be more effective if combined with immune checkpoint inhibitors affecting the PD1/PD-L1 pathway and IDO inhibitors.

In conclusion, both IFN-γ and TNF-α induce phenotype changes in MSCs. The combination of both agents results in the synergist polarization of all MSCs to a similar Th1 phenotype. Release of both IFN-γ and TNF-α by activated T cells is likely responsible for their ability to polarize MSCs to a Th1 phenotype. Since our single cell analysis showed that MSCs stimulated with IFN-γ and TNF-α cannot be divided in inflammatory and immune suppressive populations, *in vivo* studies should investigate whether the stimuli provided by MSCs activated by IFN-γ plus TNF-α results in the promotion or the reduction of the intratumoral inflammatory response. These findings also suggest that it would be worthwhile to test whether the Th1 stimulus provided by MSCs could be further enhanced *in vivo* by the concomitant treatment with IDO and/or PD-1 inhibitors.

## Materials and Methods

### Human BMSCs

BMSCs from three healthy donors were used for these studies. All the BMSCs were derived from healthy donors that met Food and Drug Administration (FDA) and AABB (formally the American Association of Blood Banks) criteria for cellular therapy donors. A protocol for the donation of marrow for BMSC production was created and registered in clinicaltrials.gov with the identification number NCT01071577. The bone marrow collection and BMSC-related research was approved by the IRB of NHLBI, NIH. Informed consent was obtained from all the donors. The experimental methods were carried out in accordance with NIH guidelines.

BMSCs were isolated from bone marrow aspirates at Department of Transfusion Medicine, Clinical Center, NIH, Bethesda, Maryland. These cells were expanded and characterized as described previously^27^. Briefly, cells from the marrow aspirates were seeded in complete media (α-minimal essential medium [α-MEM], 2 mM glutamine, 10 μg/ml gentamicin and 20% fetal bovine serum) for 24 hours and the non-adherent cells were removed. The adherent cells were expanded until 70–80% confluence was reached. Cells were sub-cultured and kept in complete media. These BMSCs were >70% viable and expressed CD73, CD90, CD105 and CD146 but not CD11b, CD14, CD19, CD34 or CD45.

### *In Vitro* Cytokine Stimulation

BMSCs (passage 4) were stimulated with 6.5 or 65 ng/ml of IFN-γ (R&D System Minneapolis, MN, USA), and with 1.5 or 15 ng/ml of TNF-α (R&D System Minneapolis, MN, USA), or the combination of IFN-γ and TNF-α (1.5 ng/ml of TNF-α + 6.5 ng/ml of IFN-γ or 15 ng/ml of TNF-α + 65 ng/ml of IFN-γ). After the adherent BMSCs were incubated with the cytokines for 48 hours, they were harvested using trypsin and were then evaluated by gene expression profiling. Prior to harvesting the BMSCs, the supernatant was collected, frozen and stored at −80 °C for protein analysis.

### Total RNA Isolation, Amplification, Hybridization and Slide Processing

Total RNA from stimulated and control BMSC samples were isolated and purified using a miRNeasyKit (Qiagen, Germantown, MD, USA). The RNA concentration was determined using a Nano Drop ND-1000 Spectrophotometer (Nano Drop Technologies, Wilmington, DE, USA) and RNA quality was assessed with an Agilent 2100 Bioanalyzer (Agilent Technologies, Santa Clara, CA, USA). RNA was amplified and labeled using an Agilent LowInput QuickAmp Labeling Kit and subsequently mixed with Universal Human Reference RNA (Stratagene, Santa Clara, CA, USA) and cohybridized to Agilent Chip Whole Human genome, 4 × 44 k slides according to the protocol provided by Agilent. The slides were incubated for 17 h at 65 °C and then the scanned using an Agilent B Scanner.

### Statistical and Microarray Data Analysis

Raw images were obtained by scanning the slides with an Agilent Scan G2505B and Agilent Scan Control software (version 9.5). The images were extracted using the Feature Extraction Software (Agilent Technologies). Partek Genomic Suite 6.4 (Partek Inc., St. Louis, MO, USA) was used for data visualization, identification of differentially expressed transcripts and hierarchical cluster analysis. We transformed the fluorescence intensity data to log2 ratios of each sample versus the universal human RNA reference (Stratagene, Santa Clara, CA, USA). Then t-tests were used to identify differentially expressed genes (both p value and FDR less than 0.05). The Ingenuity Pathway Analysis (IPA) tool (http://www.ingenuity.com, Ingenuity System Inc., Redwood City, CA, USA) was used for analysis of functional pathways. The microarray data had been deposited in GEO (GSE77814).

### Supernatant Cytokine and Growth Factor Analysis Using Multiplex ELISA

Supernatants from the BMSC samples were evaluated using a multiplex ELISA method. In total, 40 soluble factors were measured using the Bio-Plex Pro™ Human Chemokine Panel, 40-plex (Bio-Rad Laboratories, Hercules, CA, USA) and a Bio-Plex^®^ MAGPIX™ Multiplex Reader (Bio-Rad Laboratories), according to the manufacturer’s instructions. In brief, supernatant samples were incubated with microbeads coated with antibodies specific to the above-mentioned chemokines for 1 hour. After washing, the beads were incubated with the detection antibody cocktail, which included a biotinylated antibody specific to each single cytokine. After another wash step, the beads were incubated with streptavidin-phycoerythrin for 10 minutes and washed again. Finally, the fluorescence was measured using a Bio-Plex^®^ MAGPIX™ Multiplex Reader. Partek Genomic Suite 6.4 (Partek Inc., St. Louis, MO, USA) was used for data visualization and hierarchical cluster analysis. The data was normalized using log(X + offset).

### Single Cell Gene Expression Analysis

After BMSCs were incubated with IFN-γ plus TNF-α (IFN-γ: 65 ng and TNF-α: 1.5 ng) for 48 hours, they were harvested and cell sizes were measured using an automated cell counter (Countess, Invitrogen). Single cells were captured onto Fluidigm C1 IFC chip (17–25 μm) with C1 Single-Cell Auto Prep System (Fluidigm, South San Francisco, CA, USA). The single BMSCs were then lysed and the RNAs were reverse transcribed, pre-amplified and harvested using the C1 Single-Cell Auto Prep System according to the manufacturer’s instructions. Gene expression analysis of the amplified cDNAs was performed by high-throughput qRT-PCR on the Fluidigm 96.96 Dynamic Array IFC using the BioMark system. Harvested samples and primers were first loaded into 96.96 chips using a HX IFC Controller (Fluidigm) and then transferred to a BioMark (Fluidigm) for qRT-PCR reaction. Initial data analysis of the cycle threshold (Ct) values was performed with the ‘Fluidigm Real-time PCR analysis’ software and further data analysis and graphics were performed using R software and Partek Genomic Suite 6.4.

The genes evaluated by qRT-PCR were selected from the 6994 genes that were differentially expressed among unseparated BMSCs from 3 subjects stimulated with IFN-γ plus TNF-α and control BMSCs (t-tests; p < 0.05 and FDR < 0.05). Among the 6994 genes, 311 that related to IFN and TNF as determined by IPA were selected. From among these 311 genes, 2 sets of genes were selected for single cell qRT-PCR analysis; one set included 93 genes and the other 73 genes. Each set also included 3 housekeeping genes as a positive control. The set of 93 genes was made up of inflammatory cytokine and chemokine genes and the set of 73 genes was made up of genes related to Human Leukocyte Antigens (HLA), toll-like receptors and transcription factors.

## Additional Information

**How to cite this article**: Jin, P. *et al.* Interferon-γ and Tumor Necrosis Factor-α Polarize Bone Marrow Stromal Cells Uniformly to a Th1 Phenotype. *Sci. Rep.* **6**, 26345; doi: 10.1038/srep26345 (2016).

## Supplementary Material

Supplementary Table 1

Supplementary Table 2

Supplementary Table 3

Supplementary Table 4

Supplementary Table 5

## Figures and Tables

**Table 1:**
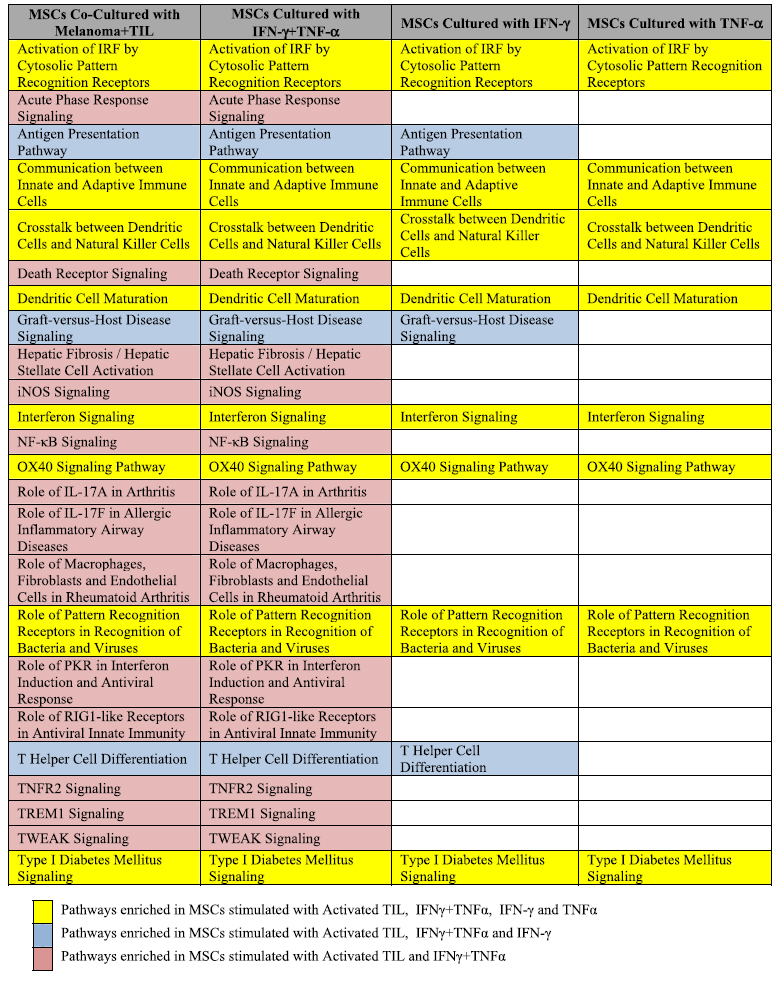
The most enriched pathways in MSCs after cytokine stimulation compared with MSCs after co-culture with activated TIL.

**Table 2:** T-cell inflamed tumor microenvironment factors expressed by stimulated MSCs.

Factor	Fold Change in Gene Expression			
	TNF-α–Stimulated MSCs	IFN-γ–Stimulated MSCs	TNF-α + IFN-γ–Stimulated MSCs	Activated TIL Stimulated MSCs
CCL2	48.5	1.8	61.9	24.4
CCL3	NC	NC	5.4	NC
CCL4	NC	NC	3.8	2.4
CCL5	31.4	4.3	70.3	20.9
CXCL9	NC	71.9	269.6	183.2
CXCL10	3.9	2.2	234.3	35.7
CXCL11	2.6	6.6	423.7	64.7
CCR5	NC	NC	NC	NC
CXCR3	1.2	1.6	2.2	2.6
STAT-1	3.2	11.3	12.5	3.9
IRF-1	3.0	13.4	15.2	11.3
T-BET	NC	NC	NC	NC
GZM	NC	NC	2.6	9.6
GNLY	NC	NC	NC	NC
IDO	−1.9	30.1	63.1	114.7
PD-L1 (CD274)	4.6	10.9	51.8	19.8
FoxP3	NC	NC	NC	NC
IL12	1,1	3.4	8.8	4.3
IL15	3.3	6.4	11.9	6.8
HLA-G	3.2	9.8	18.2	8.3

NC = No Change.

## References

[b1] Jiang X. X. *et al.* Human mesenchymal stem cells inhibit differentiation and function of monocyte-derived dendritic cells. Blood 105, 4120–4126 (2005). 15692068 10.1182/blood-2004-02-0586

[b2] Pittenger M. F. *et al.* Multilineage potential of adult human mesenchymal stem cells. Science 284, 143–147 (1999). 10102814 10.1126/science.284.5411.143

[b3] Prockop D. J. Marrow stromal cells as stem cells for nonhematopoietic tissues. Science 276, 71–74 (1997). 9082988 10.1126/science.276.5309.71

[b4] English K. Mechanisms of mesenchymal stromal cell immunomodulation. Immunol Cell Biol 91, 19–26 (2013). 23090487 10.1038/icb.2012.56

[b5] Jin P. *et al.* Direct T cell-tumour interaction triggers TH1 phenotype activation through the modification of the mesenchymal stromal cells transcriptional programme. Br J Cancer 110, 2955–2964 (2014). 24809778 10.1038/bjc.2014.235 PMC4056054

[b6] Harlin H. *et al.* Chemokine expression in melanoma metastases associated with CD8+ T-cell recruitment. Cancer Res 69, 3077–3085 (2009). 19293190 10.1158/0008-5472.CAN-08-2281 PMC3886718

[b7] Spranger S. *et al.* Up-regulation of PD-L1, IDO, and T(regs) in the melanoma tumor microenvironment is driven by CD8(+) T cells. Sci Transl Med 5, 200ra116 (2013). 10.1126/scitranslmed.3006504 PMC4136707 23986400

[b8] Wang E., Bedognetti D. & Marincola F. M. Prediction of response to anticancer immunotherapy using gene signatures. J Clin Oncol 31, 2369–2371 (2013). 23715576 10.1200/JCO.2013.49.2157

[b9] Weiss G. R. *et al.* Molecular insights on the peripheral and intratumoral effects of systemic high-dose rIL-2 (aldesleukin) administration for the treatment of metastatic melanoma. Clin Cancer Res 17, 7440–7450 (2011). 21976537 10.1158/1078-0432.CCR-11-1650 PMC3229653

[b10] Bedognetti D. *et al.* CXCR3/CCR5 pathways in metastatic melanoma patients treated with adoptive therapy and interleukin-2. Br J Cancer 109, 2412–2423 (2013). 24129241 10.1038/bjc.2013.557 PMC3817317

[b11] Ulloa-Montoya F. *et al.* Predictive gene signature in MAGE-A3 antigen-specific cancer immunotherapy. J Clin Oncol 31, 2388–2395 (2013). 23715562 10.1200/JCO.2012.44.3762

[b12] Ji R. R. *et al.* An immune-active tumor microenvironment favors clinical response to ipilimumab. Cancer Immunol Immunother 61, 1019–1031 (2012). 22146893 10.1007/s00262-011-1172-6 PMC11028506

[b13] Herbst R. S. *et al.* Predictive correlates of response to the anti-PD-L1 antibody MPDL3280A in cancer patients. Nature 515, 563–567 (2014). 25428504 10.1038/nature14011 PMC4836193

[b14] Galon J., Angell H. K., Bedognetti D. & Marincola F. M. The continuum of cancer immunosurveillance: prognostic, predictive, and mechanistic signatures. Immunity 39, 11–26 (2013). 23890060 10.1016/j.immuni.2013.07.008

[b15] Bedognetti D., Hendrickx W., Marincola F. M. & Miller L. D. Prognostic and predictive immune gene signatures in breast cancer. Curr Opin Oncol 27, 433–444 (2015). 26418235 10.1097/CCO.0000000000000234

[b16] Carretero R. *et al.* Regression of melanoma metastases after immunotherapy is associated with activation of antigen presentation and interferon-mediated rejection genes. Int J Cancer 131, 387–395 (2012). 21964766 10.1002/ijc.26471 PMC3504975

[b17] Wang E. *et al.* Prospective molecular profiling of melanoma metastases suggests classifiers of immune responsiveness. Cancer Res 62, 3581–3586 (2002). 12097256 PMC2241738

[b18] Bedognetti D., Wang E., Sertoli M. R. & Marincola F. M. Gene-expression profiling in vaccine therapy and immunotherapy for cancer. Expert Rev Vaccines 9, 555–565 (2010). 20518712 10.1586/erv.10.55 PMC3411321

[b19] Spivey T. L. *et al.* Gene expression profiling in acute allograft rejection: challenging the immunologic constant of rejection hypothesis. J Transl Med 9, 174 (2011). 21992116 10.1186/1479-5876-9-174 PMC3213224

[b20] Hodi F. S. *et al.* Ipilimumab plus sargramostim vs ipilimumab alone for treatment of metastatic melanoma: a randomized clinical trial. Jama 312, 1744–1753 (2014). 25369488 10.1001/jama.2014.13943 PMC4336189

[b21] Kaufman H. L. Combination Immunotherapy for Melanoma. JAMA oncology 1, 387–388 (2015). 26181189 10.1001/jamaoncol.2015.0479

[b22] Tumeh P. C. *et al.* PD-1 blockade induces responses by inhibiting adaptive immune resistance. Nature 515, 568–571 (2014). 25428505 10.1038/nature13954 PMC4246418

[b23] Bedognetti D. T. S., Hendrickx W., Marincola F. M. & Wang E. Toward the identification of genetic determinants of responsiveness to cancer immunotherapy. in Developments in T Cell Based Cancer Immunotherapies. (ed. S. Ascierto P. D., Wang E. ) (Springer, 2015).

[b24] Khammari A. *et al.* Adoptive T cell therapy combined with intralesional administrations of TG1042 (adenovirus expressing interferon-gamma) in metastatic melanoma patients. Cancer Immunol Immunother 64, 805–815 (2015). 25846669 10.1007/s00262-015-1691-7 PMC11029588

[b25] Dreno B. *et al.* TG1042 (Adenovirus-interferon-gamma) in primary cutaneous B-cell lymphomas: a phase II clinical trial. PLoS One 9, e83670 (2014). 24586226 10.1371/journal.pone.0083670 PMC3933342

[b26] Gajewski T. F. The Next Hurdle in Cancer Immunotherapy: Overcoming the Non-T-Cell-Inflamed Tumor Microenvironment. Seminars in oncology 42, 663–671 (2015). 26320069 10.1053/j.seminoncol.2015.05.011 PMC4555998

[b27] Ren J. *et al.* Global transcriptome analysis of human bone marrow stromal cells (BMSC) reveals proliferative, mobile and interactive cells that produce abundant extracellular matrix proteins, some of which may affect BMSC potency. Cytotherapy 13, 661–674 (2011). 21250865 10.3109/14653249.2010.548379 PMC3389819

